# Instrumental dead space and proximal working channel connector design in flexible ureteroscopy: a new concept

**DOI:** 10.1177/17562872231179332

**Published:** 2023-06-24

**Authors:** Jia-Lun Kwok, Vincent De Coninck, Amelia Pietropaolo, Patrick Juliebø-Jones, Eugenio Ventimiglia, Thomas Tailly, Florian Alexander Schmid, Manuela Hunziker, Cédric Poyet, Olivier Traxer, Daniel Eberli, Etienne Xavier Keller

**Affiliations:** Department of Urology, University Hospital Zurich, University of Zurich, Zurich, Switzerland; Department of Urology, Tan Tock Seng Hospital, Singapore; Department of Urology, AZ Klina, Brasschaat, Belgium; Endourology & Urolithiasis Working Group, Young Academic Urologists (YAU), Arnhem, The Netherlands; Progressive Endourological Association for Research and Leading Solutions (PEARLS), Paris, France; Department of Urology, University Hospital Southampton, Southampton, UK; Endourology & Urolithiasis Working Group, Young Academic Urologists (YAU), Arnhem, The Netherlands; Department of Urology, Haukeland University Hospital, Bergen, Norway; Endourology & Urolithiasis Working Group, Young Academic Urologists (YAU), Arnhem, The Netherlands; Division of Experimental Oncology/Unit of Urology, Urological Research Institute, IRCCS Ospedale San Raffaele, Milan, Italy; Endourology & Urolithiasis Working Group, Young Academic Urologists (YAU), Arnhem, The Netherlands; Progressive Endourological Association for Research and Leading Solutions (PEARLS), Paris, France; Department of Urology, University Hospital Ghent, Ghent, Belgium; Endourology & Urolithiasis Working Group, Young Academic Urologists (YAU), Arnhem, The Netherlands; Department of Urology, University Hospital Zurich, University of Zurich, Zurich, Switzerland; Department of Urology, University Hospital Zurich, University of Zurich, Zurich, Switzerland; Department of Urology, University Hospital Zurich, University of Zurich, Zurich, Switzerland; Sorbonne Université, GRC n°20, Groupe de Recherche Clinique sur la Lithiase Urinaire, Hôpital Tenon, F-75020, Paris, France; Progressive Endourological Association for Research and Leading Solutions (PEARLS), Paris, France; Department of Urology, University Hospital Zurich, University of Zurich, Zurich, Switzerland; Department of Urology, University Hospital Zurich, University of Zurich, Frauenklinikstrasse 10, CH-8091 Zurich, Switzerland; Endourology & Urolithiasis Working Group, Young Academic Urologists (YAU), Arnhem, The Netherlands; Progressive Endourological Association for Research and Leading Solutions (PEARLS), Paris, France

**Keywords:** connector design, instrumental dead space, Luer lock, stone dust aspiration, ureteroscopy

## Abstract

**Objective:**

The objective of this study was to evaluate a new concept in flexible ureteroscopy: instrumental dead space (IDS). For this purpose, various proximal working channel connector designs, as well as the impact of ancillary devices occupying the working channel were evaluated in currently available flexible ureteroscopes.

**Design and methods:**

IDS was defined as the volume of saline irrigation needed to inject at the proximal connector for delivery at the distal working channel tip. Because IDS is related to working channel diameter and length, proximal connector design, as well as occupation of working channel by ancillary devices, these parameters were also reviewed.

**Results:**

IDS significantly varied between flexible ureteroscope models, ranging from 1.1 ml for the Pusen bare scopes, to 2.3 ml for Olympus scopes with their 4-way connector (*p* < 0.001). Proximal connector designs showed a high degree of variability in the number of available Luer locks, valves, seals, angles, and rotative characteristics. The measured length of the working channel of bare scopes ranged between 739 and 854 mm and significantly correlated with measured IDS (*R*^2^ = 0.82, *p* < 0.001). The coupling of scopes with an alternative ancillary proximal connector and the insertion of ancillary devices into the working channel significantly reduced IDS (mean IDS reduction of 0.1 to 0.5 ml; *p* < 0.001).

**Conclusions:**

IDS appears as a new parameter that should be considered for future applications of flexible ureteroscopes. A low IDS seems desirable for several clinical applications. The main factors impacting IDS are working channel and proximal connector design, as well as ancillary devices inserted into the working channel. Future studies should clarify how reducing IDS may affect irrigation flow, intrarenal pressure, and direct in-scope suction, as well as evaluate the most desirable proximal connector design properties.

## Introduction

Ureteroscopy is currently the most frequent intervention for renal stones and is an established procedure commonly done in urology practice.^1^

Flexible ureteroscopy has many applications and there is a large range of ancillary and irrigation devices that can be coupled with ureteroscopes.^2^ Sometimes, there is the need for selective aspiration or injection of fluid or other medium over the working channel, usually using a connected syringe. In such cases, the column of fluid within the working channel of the scope needs to be moved. This volume can be referred to as instrumental dead space (IDS), as it has been largely studied in the field of anesthesia for lung ventilation.^3,4^ IDS may become a problem during ureteroscopy considering the low volumes available in the upper urinary tract, with mean collecting system volumes of 2.9 ml in non-hydronephrotic kidneys, and 5.1 ml with diuretic distension based on computed tomography pyelocaliceal system measurements in a recent study.^5^ Upon overinjection of fluid in a dilated pyelocaliceal system, there is a risk for overpressure and potential consecutive damage or complications such as forniceal rupture, infection, sepsis, urinoma, and hematoma.^6–9^ On the other hand, aspiration over the working channel can potentially cause damage to the mucosa if the surface is inadvertently aspirated at the tip of the scope in a collapsed pyelocaliceal system, with subsequent impairment of visibility due to bleeding.^6^ All these considerations have a potential impact on the safety and efficacy of ureteroscopy.

To date, it is not known whether there might be differences in IDS for various flexible ureteroscopes. In this present study, our aim was to evaluate the IDS of currently available flexible ureteroscopes, and the impact on insertion of ancillary devices in the working channel, as well as to review the various proximal working channel connector designs.

## Material and methods

We evaluated a series of currently available flexible ureteroscopes, including the Uscope 7.5 F PU3033A, Uscope 9.2 F PU3022A (Zhuhai Pusen Medical Technology Co. Ltd., Guangdong, China), LithoVue (Boston Scientific, Marlborough, MA, USA), (OTU Medical Inc, CA, USA), Flex-Xc and Flex-X2s (Karl Storz SE & Co. KG, Tuttlingen, Germany), as well as the URF-P7, URF-V, URF-V2 and URF-V3 (Olympus, Center Valley, PA, USA). To get as close as possible to real working conditions, the single-use scopes (Pusen 7.5 F, Pusen 9.2 F, Boston LithoVue and OTU WiScope) were new models. Reusable scopes (Storz and Olympus scopes) had all been rinsed and disinfected, passing sterilization and leakage checks after use in humans, with no record of the number of prior interventions. Scopes were evaluated in their bare state (with no removable connectors) in a straight position, in a downward 180° deflected position, then as well as with their corresponding ancillary removable connectors, if provided. Current scopes have a symmetrical deflection ability; therefore, we did not repeat the measurements for an upward deflection. All scopes with a single-opening proximal connector design in their bare state were additionally evaluated using a 3-way T-connector (Discofix C, B. Braun Melsungen AG, Melsungen, Germany).

### Instrumental dead space

IDS was defined as the volume of saline irrigation needed to inject over the proximal connector of the instrument for delivery at the distal working channel tip. Before each measurement of IDS, the working channel of scopes and any ancillary removable connectors were flushed with 50 ml of air ensuring no irrigation fluid was seen exiting the distal tip of the working channel. Then, a prefilled syringe with 2 ml saline irrigation (Injekt Luer Solo, B. Braun Melsungen AG, Melsungen, Germany) was connected to the proximal connector. If the bare scope was provided with 2 proximal Luer lock openings, the syringe was connected to the orthogonal opening relative to the working channel axis. Saline was then manually instilled until the first drop became visible at the tip of the instrument. The measured IDS was obtained by subtracting the volume left over in the syringe from its original prefilled volume. Measurements were repeated 5 times for every setup and the scope straightened between each deflection measurement.

Because IDS is related to working channel diameter and length, as well as proximal connector design and occupation of the working channel by ancillary devices, these parameters were also reviewed.

### Working channel length and estimated IDS

The working channel length was measured by using a guidewire inserted in the working channel, marking the distance between the distal tip of the ureteroscope and the proximal connector with the deflective part of the instrument held straight. Since working channel diameter may vary over the different parts of a ureteroscope, we did not measure the actual diameter but rather referred to manufacturers’ scope information brochure. This allowed us to calculate the estimated IDS based on the manufacturer’s working channel diameter and the measured length of the working channel [(diameter/2)^2^ × π × measured length].

### Proximal connector design

Photographs of the proximal connector design were taken for a descriptive evaluation. The angulation between the proximal connector and the straight axis of the scopes’ handle was measured using the computer software *ImageJ*.^10^

### Ancillary devices

Ancillary devices inserted in the working channel for evaluation of changes in IDS included: (1) laser fibers: SOLTIVE 150 µm-core (Olympus, Hamburg, Germany), FlexiFib 272 µm-core (LISA laser products OHG, Katlenburg-Lindau, Germany); (2) baskets: DAKOTA 1.9 F (Boston Scientific, IN, USA), NGage 2.2 F (Cook Medical, IN, USA); and (3) guidewires: Radifocus Guide Wire M Stiff 0.035″ (TERUMO, Leuven, Belgium), Roadrunner Hydrophilic PC 0.035″ (Cook Medical, IN, USA). We also combined the SOLTIVE 150 µm-core laser fiber and DAKOTA 1.9 F basket inserted together to simulate a clinical situation of lasering with a basket *in situ*.^11^ All ancillary devices were secured at the proximal connector using the Adjustable Biopsy Port Seal (Gyrus ACMI Inc, MN, USA), except for ancillary connectors natively including a connector seal.

### Statistical analysis

IDS was expressed as mean values. An analysis including a 3-way proximal connector setup on all scopes was performed using one-way ANOVA with Tukey post hoc comparisons. The 3-way proximal connector was either provided natively with the bare scope design, or by attachment of the according ancillary removable connector provided by each brand, respectively. Matched paired t-test analyses were performed to evaluate the impact of ancillary devices on IDS. A Pearson correlation analysis was performed to compare measured IDS with estimated IDS. For all tests, a two-sided *p* value <0.05 was considered statistically significant. All statistical tests were performed with GraphPad Prism 6.01 (GraphPad Software, La Jolla CA, USA).

## Results

### Instrumental dead space

**Figure 1.**
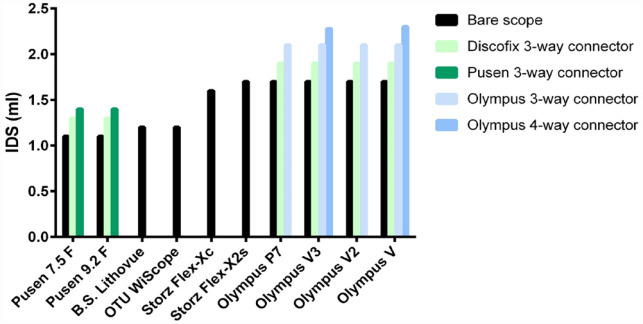
Instrumental dead space of flexible ureteroscopes.Histograms are mean values derived from the one-way ANOVA analysis.

**Figure 2.**
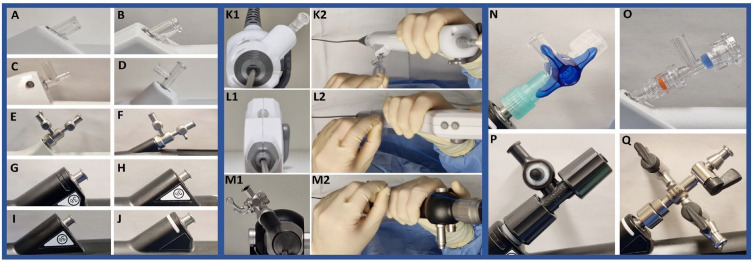
Proximal working channel designs. Proximal connector of bare scopes: A: Pusen 7.5 F, B: Pusen 9.5 F, C: Boston Scientific Lithovue, D: OTU WiScope, E: Storz Flex-Xc, F: Storz Flex-X2s, G: Olympus P7, H: Olympus V3, I: Olympus V2, J: Olympus V. Axial angle of proximal connectors with resulting hand positioning: K: −40°(WiScope), L: 0° (Pusen 9.5 F), M: +35°(Flex-X2s). *Right-hand dominant surgeon. Ancillary connectors: N: Discofix 3-way, O: Pusen 3-way, P: Olympus 3-way, Q: Olympus 4-way.

IDS was found to significantly vary between various flexible ureteroscopes, with the lowest mean IDS for a 3-way proximal connector setup (as provided by the manufacturer) in favor of the Boston Scientific Lithovue (1.2 ml) and OTU WiScope (1.2 ml), followed by Pusen 7.5 F (1.4 ml) and Pusen 9.2 *F* (1.4 ml), Storz Flex-Xc (1.6 ml), Storz Flex-X2s (1.7 ml), and finally all Olympus scopes (2.1 ml) (*p* < 0.001) (Figure 1, Table 1). The proximal connector design also largely varied between brands and scopes, partly contributing to these differences (Figure 2, Table 1). Deflection in a 180° downward position did not change the measured IDS as compared to a straight position.

**Table 1.** Characteristics of flexible ureteroscopes.

Brand	Model	Bare scopes				Scopes coupled with ancillary connectors^a^				Proximal working channel design					
		Length of the working channel (mm)	Estimated IDS (ml)	Measured IDS (ml)	Measured IDS in downward 180° deflection (ml)	Measured IDS (ml)				No. of Luer lock openings	Valves (yes/no)	Longitudinal angle between connector and handle	Axial angle between connector and 12 o’clock position of the scope	Rotative connector (yes/no)	
						Discofix 3-way connector	Pusen 3-way connector	Olympus 3-way connector	Olympus 4-way connector					3-way connector	4-way connector
Pusen	Uscope 7.5 F (PU3033A)	739	0.84	1.1	1.1	1.3	1.4	–	–	1	N	30°	0°	Yes	–
	Uscope 9.2 F (PU3022A)	741	0.84	1.1	1.1	1.3	1.4	–	–	1	N	30°	0°	Yes	–
Boston Scientific	Lithovue	812	0.92	1.2	1.2	–	–	–	–	2	N	30°	0°	No	–
OTU	WiScope	781	0.88	1.2	1.2	–	–	–	–	2	N	50°	−40°	No	–
Storz	Flex-Xc	851	0.96	1.6	1.6	–	–	–	–	2	Y	40°	0°	No	–
	Flex-X2s	854	0.97	1.7	1.7	–	–	–	–	2	Y	30°	+35°	No	–
Olympus	URF-P7	844	0.96	1.7	1.7	1.9	–	2.1	–	1	N	40°	0°	Yes	–
	URF-V3	835	0.95	1.7	1.7	1.9	–	2.1	2.3	1	N	40°	0°	Yes	Yes
	URF-V2	835	0.95	1.7	1.7	1.9	–	2.1	–	1	N	40°	0°	Yes	–
	URF-V	833	0.94	1.7	1.7	1.9	–	2.1	2.3	1	N	40°	0°	Yes	Yes

a Except for scopes natively provided with a fixed 3-way connector.

### Proximal connector design

Proximal connector design analysis revealed that all scopes had at least one Luer lock opening in their bare state, with the Flex-Xc, Flex-X2s, Lithovue, and WiScope being natively provided with a fixed 3-way connector design, resulting in 2 orthogonal Luer lock openings (Figure. 2). These 2 Luer locks were natively equipped with a valve in Storz Flex-Xc and Flex-X2s scopes. The Pusen scopes were provided with an ancillary 3-way connector without valves, which had a port seal at the opening in line with the working channel. The Olympus P7, V, V2, and V3 were provided with an ancillary 3-way connector by the manufacturer, with the V and V3 including an additional ancillary 4-way connector. All these ancillary Olympus connectors had valves at the Luer lock, except for the 3-way connector where the opening in line with the working channel was equipped with a seal port. Once attached, all ancillary removable connectors were found to rotate freely around the Luer lock of the bare scope.

The longitudinal angle between the proximal connector and handle differed between scopes, ranging from 30° to 50° (Figure 2, Table 1). Two scopes had proximal connectors not in-line with the axial angle between the connector and 12 o’clock position of the scope, with the Storz Flex-X2s being +35°, and the OTU WiScope being −40°, referenced to viewing the scope from the proximal end.

### Working channel

**Figure 3.**
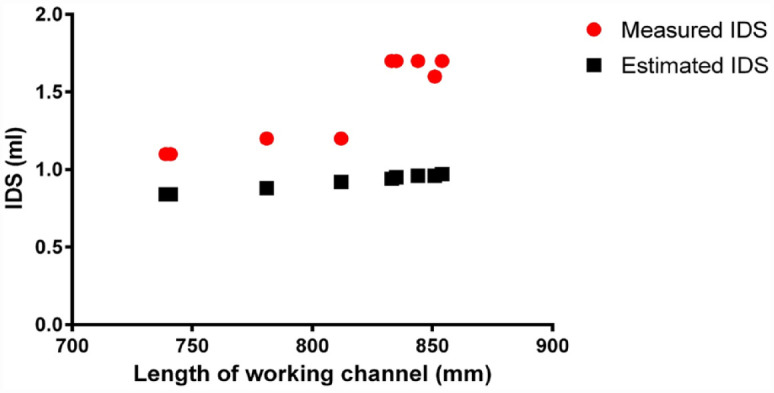
Correlation between IDS and length of working channel.

The measured length of the working channel ranged between 739 and 854 mm (Table 1), and significantly correlated with measured IDS (*R*^2^ = 0.82, *p* < 0.001; Figure 3). The diameter of the working channel was invariably 3.6 F, according to manufacturers’ brochures. The resulting estimated IDS ranged between 0.84 and 0.97 ml.

### Devices resulting in IDS reduction

The use of the Discofix 3-way connector instead of the 3-way connector provided by the manufacturers resulted in a mean IDS reduction of 0.2 ml [95% confidence interval (CI) 0.1–0.2; *p* < 0.001; Table 2]. An IDS reduction was also found when ancillary devices were inserted in the working channel, ranging from 0.1 ml for laser fibers to 0.5 ml for guidewires (Table 2).

**Table 2.** Devices resulting in IDS reduction.

	Mean IDS reduction (95% CI)	Change^b^	*p* value
Discofix 3-way connector^a^	0.2 ml (0.1–0.2)	9%	<0.001
Olympus Soltive 150 µm fiber	0.1 ml (0.1–0.1)	6%	<0.001
LISA FlexiFib 272 µm fiber	0.1 ml (0.1–0.2)	9%	<0.001
Boston Scientific Dakota 1.9 F basket	0.3 ml (0.3–0.3)	17%	<0.001
Cook N-Gage 2.2 F basket	0.4 ml (0.4–0.4)	23%	<0.001
Terumo Stiff 0.035″' guidewire	0.5 ml (0.5–0.6)	32%	<0.001
Cook Roadrunner 0.035″ guidewire	0.5 ml (0.5–0.6)	32%	<0.001
Olympus Soltive 150 µm fiber + Boston Scientific Dakota 1.9 F basket	0.4 ml (0.4–0.5)	25%	<0.001

IDS, instrumental dead space; CI, confidence interval.

a Analysis was limited to scopes that were not provided with a fixed 3-way connector (Pusen scopes and all Olympus scopes).

b Percent change is relative to overall IDS.

## Discussion

To the best of our knowledge, this is the first study available in the literature evaluating IDS in flexible ureteroscopy. The study reveals that IDS varies significantly between different flexible ureteroscope models, ranging from 1.1 ml for Pusen bare scopes, up to 2.3 ml for Olympus scopes coupled with their 4-way connector. Deflection of the scopes 180° downward did not change the measured IDS. The coupling with an alternative ancillary proximal connector or the insertion of an ancillary device into the working channel was found to significantly reduce IDS.

The findings of the study are of relevance for clinical practice since injection and aspiration through the working channel are frequently performed for various indications during ureteroscopy (Table 3).^11–22^ An IDS as low as possible is desirable in such situations since the mean intrarenal volume is estimated to range between 3 and 5 ml in non-hydronephrotic human kidneys.^5^ Considering this, the IDS of the evaluated flexible ureteroscopes represents 22% to 77% of the intrarenal volume. In summary, the lower the scope IDS, the better the scope performance in such clinical scenarios (Table 3). Aspiration of urine for cytology or microbiology exemplifies the clinical impact of IDS, since the first 1–2 ml of aspirate will likely not be representative of fluid from the tip of the scope, and further aspiration may cause mucosa entrapment at the tip of the scope possibly causing bleeding complications.^6^ Conversely, overinjection to overcome IDS may be associated with the risk of diluting specimens or causing high intrarenal pressure, which in turn is associated with serious hazards such as bleeding and infection.^23^

**Table 3.** Ureteroscopy clinical practice involving dead volume shifts.

Aspiration	Injection
Urine for cytology/Barbotage	Contrast medium
Urine for microbiological workup	Methylene blue: Blue spritz test
Stone dust aspiration (active or passive)	Air bubbles: Orientation purposes (goes anteriorly)
Air bubbles	Blood clots: Subsequent stone dust evacuation with a basket
Blood clots	Saline: Syringe or hand-assisted irrigation device post instillation of contrast or methylene blue
Blood-contaminated saline to clear the view	
Fibrous or matrix deposits	
Evacuation of contrast medium	
Evacuation of methylene blue	

While the diameter and length of the working channel of scope are fixed and may not be adjusted by the operator, ancillary devices placed within the working channel may favorably result in IDS reduction. This advantage may typically be exploited for stone dust aspiration over the working channel.^16,17,24,25^ Stone dusting capabilities with newer lasers have been improving^26,27^ and the role of stone dust aspiration will likely expand in the years to come, thus underlying the clinical significance of the findings of this study.^28^ The laser fiber occupying the working channel presents an additional advantage for stone dust aspiration, as the fiber may be gently inserted and retrieved in the case of trapping stone dust in the working channel. In our experience, this technique alleviates the need for instrument retrieval and time-consuming cleaning of the working channel. Larger inserted ancillary devices such as guidewires may reduce IDS to an even greater extent, but might become disadvantageous because of the negative impact on irrigation flow,^29,30^ as well as the resultant lower size limit of stone dust that can be aspirated over the working channel.^16,31^ Another workaround alleviating the limitations associated with IDS and stone dust aspiration over the scope would be to use a dual-channel ureteroscope, with one channel dedicated to continuous irrigation inflow and the second working channel for continuous or intermittent aspiration.^24,32,33^

For scopes with a single Luer-lock proximal connector design and removable ancillary proximal connectors, the overall IDS may be lowered by a mean 0.2 ml using a Discofix 3-way connector instead of the natively provided connectors, according to the results of this study. The Luer lock in line with the working channel may be equipped with a port seal, as aforementioned in this study. One must beware of this technique representing an off-label use of the Discofix and bears a serious risk of unintentional laser fiber breakage within the 3-way T-connector if the T-knob is unintentionally rotated to stop irrigation.

We calculated the estimated IDS based on working channel size specifications cited by the manufacturers and considering the measured working channel length. Interestingly, the estimated IDS was systematically lower compared to the measured IDS (Table 1, Figure 3). Also, the differences in working channel length were only partially explained by differences in measured IDS (*R*^2^ = 0.82). Deflecting the scope did not impact the IDS either, reminiscent of prior studies where irrigation was affected negligibly to none by deflection of scope.^29,30^

The discrepancy between estimated and measured IDS may be explained by the working channel caliber likely not being uniform throughout the scope. This is a reminder that the quoted working channel caliber by manufacturers is usually the minimum caliber of the working channel and may become larger in some sections along the whole working channel path.

For all scopes included in the present study, the working channel length was found within a rather narrow range, with only a 13% difference between the shortest and longest length (739 *versus* 854 mm). Scope manufacturers supposably have chosen a working length long enough to fit all patients’ sizes. Arguably, one may easily reduce IDS if customized flexible ureteroscopes with shorter working channels were available, reminiscent of the concept of varying DJ-stent lengths according to patients’ characteristics.^34,35^ Just recently, a novel female flexible ureteroscope with a shorter working length was introduced on the market and presents an opportunity for reducing IDS.^36^ Single-use scopes may be of particular interest allowing a larger range of customized scopes readily available in the future.

The concept of IDS can be applied widely in endourology across various instruments: for example, all fluoroscopy operations involve the injection of contrast medium with dead space shifts. Going beyond ureteroscopes, IDS can be applied to the majority of instruments routinely used for injection or aspiration in urology, including cystoscopes, ureteral catheters,^37,38^ nephroscopes, and nephrostomy tubes.^39,40^ A greater impact would likely be in instruments used in the upper urinary tract, due to the smaller volume of the pyelocaliceal system compared to the bladder. This may also have an impact on the methods of instillation of upper urinary tract carcinoma topical therapy agents^38^ with differing volumes needed to overcome the instrument dead space, particularly in periods of worldwide shortage.^41,42^

Prior studies have evaluated ureteroscope parameters such as irrigation flow,^29,30^ direct in scope suction,^17^ intrarenal pressure,^43,44^ and dust particle size aspirated.^16,31^ How IDS impacts these parameters is of high interest and certainly deserves future evaluation in separate studies. We believe that the inference of any sizable measures will not only depend on the IDS per se, since slight differences in working channel size, length, and shape^30^ may widely impact parameters such as flow and suction with consequences on intrarenal pressure and dust particle size aspiration. This is even more valid when a device such as a laser fiber is inserted in the working channel.

The analysis of proximal connectors design revealed a high degree of variability in the number of available Luer locks, valves, seals, angles, and rotative characteristics. A minimum of 2 Luer locks seems desirable to couple the scope with irrigation and ancillary devices into the working channel together. Likewise, a valve on the irrigation port seems desirable to control irrigation flow. Conversely, a seal port in line with the working channel seems desirable to waterproof any inserted ancillary devices. The longitudinal angulation of the proximal connector with the handle was found within a narrow range (30°–50°), and the axial angle of the proximal connector was in line with the 12 o’clock position in most scopes, which both seem adequate for most applications. Only two scopes – the OTU WiScope and the Storz Flex-X2s – had a proximal connector axial angle not in line with the 12 o’clock position. These setups may positively or negatively impact on handling of the scopes and insertion of ancillary devices into the working channel. For example, when the Flex-X2s is held in the right hand, the left hand needs to cross hands to reach beyond the axis of the scope for access to the Luer lock (Figure 2M). Conversely, the OTU WiScope has a proximal connector located closer to the torso of the surgeon, which may cause inserted ancillary devices to travel toward the face of the surgeon, with its inherent sterility hazards (Figure 2K). Finally, a fixed proximal connector seems more favorable than a rotative one since the surgeon would constantly need to check the position of the rotating Luer lock when manipulation is needed at that level during the surgery. Arguably, all the above advantages and limitations are affected by surgeons’ positioning habits,^11^ and may largely vary according to surgeons’ preferences.

This study has several potential limitations. The syringe used for measurements was a 2-ml syringe with 0.1 ml graduation. The authors believe that measurements of less than 0.1 ml are not clinically relevant and would therefore not impact the study findings. This would also apply to eventual superficial coating damage of the working channel in reusable scopes. More serious coating damage would cause leak test failure and such scopes would therefore not have been used in the present study. Each scope model was represented by only one single scope, respectively, with possible small differences from scope to scope in the same model not being accounted for. However, all experiments were repeated 5 times for each setup and scope to reduce the risk of intra-scope measurement discrepancies.

## Conclusion

IDS appears as a new parameter that should be considered for future applications of flexible ureteroscopes. A low IDS seems desirable for several clinical applications. Surgeons should be aware of different IDS for different scopes, and that use of a scope with shorter working channel, alternative proximal working channel connectors or insertion of ancillary devices may reduce IDS. Future studies should clarify how reducing IDS may affect irrigation flow, intrarenal pressure and direct in-scope suction, as well as evaluate the most desirable proximal connector design properties.

## Declarations

**Ethics approval and consent to participate:** Not applicable.

**Consent for publication:** Not applicable.

**Availability of data and materials:** Raw data on the experimental setup are available from the corresponding author on reasonable request.
